# Modern Sociology

**Published:** 1903-01-24

**Authors:** 


### MODERN SOCIOLOGY.

### THOUGHTS ON METHODS OF CHARITY.

BY A CORRESPONDENT.

It is rather strange that out-door relief should so often be granted to paupers, and anything akin to it refused to those who are above the rank of paupers, yet need aid from outside sources. It is considered hard that a poor person whose life has not been absolutely disreputable should be offered the "house," but the "house" is offered without hesitation to well-doing persons, not absolutely destitute, whom distress or infirmity prevents being wholly self-supporting. It is true that in this case the workhouse is called the "asylum," or the "home," but, in fact, it is simply a question of indoor relief as opposed to the outdoor kind. Why should a blind man be sent to a blind asylum? He may reasonably go to a school for the blind, where he can be taught some employment by which he may in part, if not altogether, earn his living; but why should he pass his whole life there? And why, if he returns to his home again, should he not receive from the charity which maintains the asylum such a sum as will prevent his being entirely a burden to his relations, who are perhaps unable to maintain him in idleness. Nay, if he has been taught a trade, why should the asylum charity not arrange to supply him with work which he can do, or at least negotiate its sale for him, without insisting on his remaining in a colony of persons similarly afflicted to himself. All afflicted people need kindly handling, but they do not need to be among others placed exactly like themselves, nor is it good for them to be so. Their misfortune isolates them from their fellows quite enough, without adding any artificial isolation of environment or residence. For lack of exercise, as well as from the sense of deprivation, blind people are apt to be melancholy, and therefore it is better for them to live as much as possible with those who are in the full tide of activity, and who will bring them in touch with all they can of life. The knowledge that at best they must be excluded from so much of both work and pleasure may cause an occasional touch of repining, but even that is healthier than the dulness and stagnation which must arise when all their companions are their fellows in misfortune. To instance the grandest of blind men whom this generation remembers, would it have been a good thing for Henry Fawcett or for the nation he belonged to if his life had been passed within the walls of an asylum for the blind? He had means to save himself from such a misfortune, and let himself be shut out from neither intellectual, social, nor public life on account of it. Why should not others, in their degree and according to their power, be allowed as well to feel that their deprivation of one sense does not put them outside the pale of ordinary society.

The same thing holds good of deaf mutes. Let us by all means have schools for the training of deaf mutes, and let that training be as good as possible. Let them be taught speaking and lip-reading—an education which must needs be expensive, and which therefore their relations cannot always afford. There absolute charity may come in with advantage. But once taught, they should be encouraged to go back to their homes and seek work in the outer world. No one who has met intelligent deaf mutes and noted with surprise how little of even ordinary conversation they miss when they can see the speaker, will doubt that many of them will become entirely self-supporting. But to others a little grant-in-aid might make all the difference between misery and comfort. Is there any reason why charity should not give it?

In many cases of incurable disease also it would be better that the patient should not live among other invalids. Acute disease is sufficiently selfish to make the sufferer comparatively indifferent to the death of the patient in the next bed, but chronic disease is a different thing. In an ailment such as epilepsy, where the attacks may follow each other at irregular intervals during the course of a long life, is it necessary that the patient should live in an epileptic colony? These colonies have proved useful, because the patients there have been set to work that is healthy, and had their general course of life carefully regulated. It is not the fact that they are in a colony that of itself has benefited them. Indeed, one would like to know if the sight of a companion in a fit has not sometimes affected others sympathetically. But, above all, when the only misfortune is that sufficiently fell one—poverty—why should the sufferer be removed from the familiar home, and see the faces of friends and kinsfolk only now and then? Why, above all, should the man who has grown old and past work be sent so often to a place where life's failures in the same trade are housed? Why should the old shoemaker be sent to the "Decayed Cobblers' Home," and the baker, who will send out loaves no more, to the "Bakers and Flour Merchants' Benevolent Institution"? When they were working, these men did not associate exclusively with those of their own trade. Why should they do so at the end of their days? They are none the happier for it. It is quite comprehensible that a man who has amassed wealth in a certain pursuit should want especially to help those in the same business who have been less fortunate than he; but there is no need that his generosity should take the form of a large building, or even a set of cottages where the recipients of his bounty may be housed. Of course the big building which can be visited by subscribers is an actual proof of money given, and an obvious reason, in the expense of its upkeep, for asking for more; but it does not follow that the money subscribed is thus put to the best possible use. It is rather a pleasant trip, too, for the committee to pay the place an annual visit, and admire the spotless rooms and corridors—ah, so chill in their spotlessness sometimes!—look out on the well-kept gardens, and feel a glow of conscious generosity warm their beings. What do they know of the dulness of the winter evenings, the lack of cheerful companionship, the feeling that one has no longer a place in the world, which adds so much to the bitterness of old age?

We might speak also of the even greater unwisdom of bringing up young people in institutions, but though that may be even more cruel in the end, at least the victims do not at the time feel it to be so. But sensitive souls shrink from such an avowal of poverty, even as the best of a humbler class shrink from the workhouse. Nay, many of them will not accept it. The little pension, given quietly, almost surreptitiously, that would enable them to keep a tiny home over their heads, would be welcome, but they shrink from that going around and making parade of their poverty which is an inevitable part of accepting institutional hospitality. Indeed, those who need help most cannot obtain it, for in most of these places the voting system still obtains. Not those who need help most, but those who have most friends to work for them, have the best chance of success, for votes are given oftentimes not so much because the voter is convinced of the necessity of the case, as because he wishes to oblige the person who asks him for his vote for a particular candidate, and from whom, in turn, he can ask some future favour. The whole system is vicious, and as a charity, futile. The good which it does bears no proportion to the cost and trouble it entails, and, as a rule, it fails altogether to reach the most deserving.

